# The Steam Gauge for Vulcanizing

**Published:** 1865-09

**Authors:** 


### THE STEAM GAUGE FOR VULCANIZING.

We have used, for several months past, the steam gauge for vulcanizing, as first suggested by Dr. A. Lawrence, of Lowell, Mass. The results obtainable in its use have been more uniform and satisfactory than by the use of the thermometer. Why it is so, it might seem d fficult at first to define; but the gauge si far less liable to injury than the ordinary thermometer, and the gauge all the while gives a true indication of the amount of steam. The thermometer may become injured so as to destroy its accuracy, and yet that injury be imperceptible to ordinary observation.

All who use vulcanizers are aware that sometimes they have found it difficult to vulcanize at all. This is usually dependent upon the imperfect condition of the thermometer. The slightest, and almost imperceptible, check in the bulb of the thermometer, will occasion such trouble. Nothing of the kind can occur with the gauge. Should it become injured, the fact would be apparent, for it would cease to operate. It may be used for years. Thermometers are very frequently broken; much, of course, depends upon the care of the workmen.

The cost of the gauge in the beginning seems a large item; but in the end will be found to be economy to those who are in constant use of the vulcanizer.

Dr. Lawrence has given a full discription of the gauge, and the method of using it, in former numbers of the register, to which we will refer the reader.

